# Gender, health and ageing in Fiji: a mixed methods analysis

**DOI:** 10.1186/s12939-021-01529-9

**Published:** 2021-09-14

**Authors:** Rebecca Dodd, Janani Shanthosh, Thomas Lung, Aporosa Robaigau, Mai Ling Perman, Eric Rafai, Roslyn Poulos, Anthony B. Zwi, Renu John, Anna Palagyi

**Affiliations:** 1 The George Institute for Global Health, University of New South Wales, 1 King Street, Newtown, Sydney, NSW 2042 Australia; 2 Faculty of Medicine and Health, Sydney School of Public Health, The University of Sydney, Sydney, NSW Australia; 3 School of Medical Sciences, Fiji National University, Suva, Fiji; 4 Ministry of Health and Medical Services, Suva, Fiji; 5 School of Population Health, University of New South Wales, Sydney, Australia; 6 School of Social Sciences, University of New South Wales, Sydney, Australia; 7 The George Institute for Global Health, Hyderabad, Telangana State India

**Keywords:** Ageing, Gender, Health systems, Fiji, Pacific Islands

## Abstract

**Background:**

Women are disadvantaged by ageing: older women are more likely than older men to suffer from ill-health, have less access to health care and suffer discrimination within the health care system. Globally, there is a dearth of health research on gender and ageing with substantial knowledge gaps in low and middle-income country contexts. Part of a wider investigation on health and ageing in Fiji, our objective was to identify and describe gendered differences in healthy ageing in this Pacific Island context. We believe this to be the first such study in the Pacific region.

**Methods:**

Applying a health systems lens, we used a mixed-methods approach, encompassing analysis of cause of death data; focus group discussion to gather community and family attitudes to health services; and policy analysis, and then used data triangulation techniques to draw out key themes and insights.

**Results:**

We found that gender affects health outcomes among older persons, attitudes towards and experience of healthy ageing, and an older person’s access to and use of health services. We also found that while Fiji’s policy response to ageing has recognised the importance of gender, to-date there has been limited action to address gender differences. Gender (as oppose to sex differences) has direct and indirect implications for the health of older Fijians, while gendered *inequalities* and patriarchal norms appear to affect both men and women’s experience of ageing and the health system response. Further, gender and age discrimination may be intersecting, intensifying their separate effects.

**Conclusion:**

This study demonstrates the feasibility and importance of applying a gender lens to the study of healthy ageing. Our findings from Fiji may be relevant to other island nations in the south Pacific which share similar challenges of population ageing, a constrained health budget and geographically-dispersed populations. The data triangulation methodology may be considered an efficient and insightful way to examine gendered responses to healthy ageing elsewhere.

**Supplementary Information:**

The online version contains supplementary material available at 10.1186/s12939-021-01529-9.

## Background

The World Health Organization (WHO) defines healthy ageing as “*the process of developing and maintaining the functional ability that enables wellbeing in older age*,” [1] noting that functional abilities are determined by an individual’s intrinsic capacities, the environment in which they live and the interactions between them. Gender and gendered discrimination -- the result of social and cultural norms, customs and stereotypes, as well as legal, political and economic constraints on the advancement of women [2] -- represent an important part of this environment and have a significant impact on men and women’s experience of the ageing process.

Older women are more likely than older men to suffer from ill-health, have less access to health care and suffer discrimination within the health care system [3–5]. These inequities persist despite women enjoying longer life expectancy than men in many settings [3] and men suffering worse health outcomes from certain conditions – such as cardiovascular-related mortality [6, 7]. Globally, older women are more likely than older men to be living with a functional disability (defined as difficulty in performing personal care activities including basic activities such as eating and washing) [4, 8]; to suffer from Alzheimer’s disease and dementia [9, 10]; less likely than men to have access to timely and appropriate investigations and treatment for heart disease and stroke [11, 12]; and women aged 50 years or older have a four times higher rate of osteoporosis and a two times higher rate of osteopenia compared with men [13]. Older women are more likely than older men to be anxious or depressed [3, 4], to be poorer [14] and to suffer from elder abuse [15, 16].

As women age, health costs consume a greater proportion of their income [3] and many lack resources for transport to health services, provider fees and medicines. Lower literacy levels and social norms mean women are less empowered in health decision-making [14], more likely to be alone in old age and to suffer from social isolation [4].

Many Pacific Islands still have a predominantly young population structure, however – as elsewhere in the world – their populations are ageing rapidly [17, 18]. This demographic transition will inevitably increase pressure on health systems stretched by a high burden of non-communicable diseases [18]. Especially in the South Pacific, health care already consumes a large proportion of government budgets, leaving limited financial scope to expand services for the growing population of older persons [19]. Pacific island countries also share operational health system challenges related to ensuring equitable provision of health care to small, widely-dispersed populations [20]. Understanding the health aspects of ageing in the Pacific, including the different needs and experiences of men and women, will help ensure services are appropriate and effective, making best use of health budgets. WHO notes a dearth of health research on gender and ageing globally [3] and this is also true in the Pacific: little is known on how ageing is affecting the health of Pacific men and women [18, 21], and their access to health care including end of life care [22].

This study contributes to filling the Pacific knowledge gap through an analysis of gender and healthy ageing in Fiji. The most populous Pacific nation after Papua New Guinea, Fiji is a small country of 880,000 people [23] with the population dispersed over 300 islands. A middle-income country, Fiji has good human development outcomes with a Human Development Index (HDI) ranking of 0.743, which is considered ‘high’ [24]. Fiji’s neighbours Kiribati, Samoa, Tonga and Vanuatu have similar HDI rankings, falling between 0.609 and 0.725 [25]. While Fiji’s overall population profile is young, with a median age of 27.5 years, it is rapidly ageing and this has a gendered aspect: women account for 51.4% of those aged 60–64 years, but almost 60% of those over 75 years [23]. Fiji suffers from deep gender inequities: despite growing gender parity in access to education, women’s annual earnings are less than half those of men, US$8300 compared to $17,500; and 64% of women have experienced intimate partner violence [24, 26, 27]. UNDP reports that gender inequality remains pervasive in many Pacific societies [28] while the UN Population Fund (UNFPA) has noted the need for further analysis on older persons in Fiji, and in particular, older women [17].

Fiji’s health system is predominantly publicly-financed and delivered free or at very low cost; older persons are entitled to free care for most procedures. An estimated 70–80% of the population has access to primary health care, delivered mainly by community health workers and nurses [29, 30].

Part of a wider investigation on the health system response to ageing in Fiji, this study aimed to identify and describe the gendered dimensions of healthy ageing in that country. Given the dearth of studies on gender and ageing in the Pacific – we believe this to be the first – we took a mixed-methods approach, incorporating analysis of mortality data, community and family attitudes to health services and policy review. Comparing qualitative and quantitative data can generate new insights, particularly on complex health questions [31]. By touching on disparate aspects of the ageing experience we aimed to form a multi-dimensional picture of the influence of gender on lived experiences and outcomes. Further, we sought to demonstrate the relevance and feasibility of a gender analysis that combined different methodologies commonly used in healthy ageing research.

## Methods

Our approach was informed by literature on health sector gender analyses [32, 33], which emphasizes the need to move beyond empirical data to understand *how* access to resources, social norms, rules and decision-making *are gendered*, and how these in turn influence health care and health seeking behaviour [32]. Within these broad categories, the following areas of investigation have been suggested as providing insight into gender dimensions of health and health systems: social determinants of health including poverty, social status and literacy and how these differ between men and women; differences in access to care among men and women – including access to transport; perceptions of care quality; and, the extent to which policies guiding health service delivery are gender responsive, and women are represented in decision making bodies [32].

Based on this guidance, we identified three research questions which aimed to fill basic knowledge gaps while also providing an holistic understanding of gender differences in healthy ageing in Fiji:

- How does mortality differ between older men and older women, and can these differences be attributed to gender?
- What are key differences in women’s and men’s experience of healthy ageing?
- Has the health sector response to healthy ageing to date been gender sensitive?

Data analysis methods that met the objectives of the broader study and were also conducive to gender analysis were undertaken, namely: a cause of death analysis; a qualitative study of community views of ageing; and, a policy analysis of national policies and strategies underpinning Fiji’s response to population ageing. Data were collected in Fiji between June 2019 and March 2020. Methods and results for each workstream are presented below, while the discussion draws out key themes and insights from across the three areas of investigation.

We have used the broad age group of 55 years and over to represent ‘older adults’ in Fiji. While this is below the more-commonly used 60 or 65 years, the relatively young average life expectancy in Fiji of 67 years [34], compulsory retirement age of 55 years for public servants and cultural perceptions of ‘elders’ suggest a ‘younger’ cut-off age may be appropriate.

### Cause of death analysis methods

The cause of death analysis was conducted as part of the broader study on health and ageing in Fiji, and relevant gender findings included in this study. An ecological time-series study of mortality trends in adults aged 55 years and over was conducted in Fiji over the period of 2008–2017 using the Fijian Civil Registration and Vital Statistics (CRVS), the national database of cause-of-death registration coded according to the WHO’s 10th revision of the International Statistical Classification of Diseases and Related Health Problems [35]. Age-standardised mortality rates (ASMR) per 100,000 population were calculated annually using the Fay-Feuer method [36] by sex, place of death and for the six leading causes of death. Direct age-standardisation to the Fijian population was calculated using the 2017 Population and Housing Census [23]. Population numbers and place of death by the 14 provinces were placed into three categories: maritime (Kadavu, Lau, Lomaiviti and Rotuma), rural (Bua, Cakaudrove, Nadroga, Navosa, Ra, Serua-Namosi and Tailevu) and urban (Ba, Macuata, Naitasiri and Rewa). The Joinpoint Regression program was used to estimate the average annual percentage change in ASMRs between 2008 and 2017 [37].

### Focus group discussion methods

The methods for the focus group discussion (FGDs) are described in detail elsewhere [21]. In brief, we used data from 19 FGDs, each comprised of 4–10 persons (older persons and family members) including eight female-only focus groups, seven male-only focus groups and four mixed-gender groups. FGDs were held in rural and urban areas, and each was conducted in the local language of participants’ choice (English, iTaukei or Fijian-Hindi) and led by a moderator experienced in community consultation in Fiji, who was bi-lingual in English and the local language and trained in interview and group facilitation techniques. The moderator was supported by a separate observer and note taker. Focus groups were performed separately for iTaukei and Indo-Fijian community members and were moderated by a person of the same sex and ethnicity. Focus groups were audiotaped and lasted approximately one hour. A summary of the discussion was read back to participants at the completion of each focus group in order to confirm the interpretations of the participants’ responses. Basic demographic details of focus group participants were elicited by a short, interview-based questionnaire.

Data analysis occurred in two stages. In stage one, transcripts were separately coded by two members of the research team and codes reviewed, refined and sorted into a single coding framework based on main categories of discussion. Emerging themes were then mapped to the COM-B (Capability, Opportunity, Motivation, Behaviour) model – the central hub of a broader behaviour change analytical model [38], selected to facilitate the systematic and transparent identification of policy categories and intervention functions that may support behaviours to promote healthy ageing. The model has three components:

- Capability: physical or psychological ability of men and women to achieve healthy ageing.
- Motivation: unconscious or automatic mechanisms that activate or inhibit behaviours, including habitual and emotional responses, as well as analytical decision-making.
- Opportunity: how the social and physical environment enables (or restricts) healthy ageing [38].

In stage two, thematic content from male, female and mixed groups were compared, i.e., all data coded to ‘capability’ and its associated themes from male-only focus groups was compared to the same data from female-only groups. This exercise was repeated for the ‘motivation’ and ‘opportunity’ components. We identified the themes that generated most discussion, and then looked for similarities and differences in relation to each theme that might constitute gender differences in the experience of healthy ageing. The management and analysis of qualitative data was supported by NVIVO 12 software (QSR International).

### Policy analysis methods

The policy mapping exercise was carried out in two stages. First, we conducted a comprehensive search for Fijian Government policies, strategies, decrees and other official documentation relevant to: health service provision; aged care; gender; disability; financial protection; social security and pensions. This included searching for national policies and plans for health, disability and gender as well as relevant economic and social policies, for example on social care, violence against women and pensions. Documents were sourced through on-line searches using Google, complemented by discussion with relevant stakeholders including staff at the Ministry of Health and Medical Services who advised on (and where necessary provided) the appropriate documentation for review. To understand the policy context, we reviewed the grey literature for reports and analyses on demographic change, ageing, health systems and services and gender issues in Fiji; grey literature evidence was drawn on in the discussion but not incorporated into the policy analysis.

We then scanned these documents for policy commitments or planned actions relevant to ageing and gender by conducting a free word search for “health” and/or “older/ageing/elderly/senior” and/or “women/men/ female/male/ gender”. Contents lists, headings and sub-headings and references were scanned for additional, relevant information. Text on policies and associated actions was extracted and tabulated. Background information, e.g. on the living conditions of older women, was noted but not extracted, given our intention to focus on and analyse policy commitments.

### Data triangulation

We followed a recognized approach to mixed-methods data analysis: first analysing each set of results separately, then looking across the three data sets to identify common, contrasting and/or complementary themes [31, 39]. Through data triangulation we aimed to form a multi-dimensional picture of gender issues relevant to healthy ageing, using different sources to ‘triangulate’ our emerging findings (‘data’) and provide these with explanatory depth. Denzin describes the praxis of triangulation as a “dialectical process” which can provide a more nuanced understanding of research findings, and place disparate findings in dialogue with one another, while Greene et al. note that triangulation can serve the goal of “complementarity” by revealing different facets of understanding [40, 41]. Hesse-Biber argues that triangulation is an appropriate methodology for analyses, including feminist analysis, aiming to “uncover subjugated knowledge.” [42] Similarly, Flick suggests that triangulation “gives access to different versions of the phenomenon that is studied” facilitating greater insight [43].

## Results

### Cause of death analysis

Disaggregating cause of death data in Fiji revealed important differences in mortality trends between men and women at the national level, and populations living in rural, urban and maritime areas. A full report of cause of death in Fiji is found in Palagyi et al. [44]; here we draw out some salient examples from that analysis which highlight key gender differences.

The top six causes of death in adults aged 55 years and over were the same for men and women in the 10-year period from 2008 to 2017, however there were important gendered differences in the burden of each disease. Men suffered a higher mortality rate for diseases of the circulatory system; respiratory illness; and, infectious and parasitic disease: for example, in 2017 the ASMR from respiratory diseases for men aged 55 and over was 230 (95% Confidence Interval [CI] 192–272), almost double that of women of the same age, 120 (95%CI 95–272). By contrast, women experienced a higher burden of neoplasm deaths in every year of the ten-year period of analysis: with an ASMR of 470 (95%CI 418–536) in 2017, compared to 367 (95%CI 319–419) for men. See supplementary file 1.

Disaggregation of data by geographic area also revealed key gender differences (see Table 1). For example, there was a statistically significant *decrease* in the average annual percentage change in ASMR for diseases of the circulatory system in maritime areas – a key finding in and of itself – but the annual rate of decline for women (8.9% (95%CI 3.7–13.7)) was almost double that for men (4.6% (95%CI 0.1–8.8)), between 2008 and 2017. By contrast, in rural areas, the average annual percentage change in ASMR for neoplasms increased at a statistically significant level (*p* < 0.05) by 11.7% (95%CI 6–17.7) annually for men, compared to a 6.3% (95% CI -0.1 – 13.1) increase for women, which was not found to be statistically significant.

**Table 1:** Age-standardised mortality rates (per 100,000) and annual percentage change for cardiovascular disease, diabetes and cancer in Fijian males and females aged 55 years and over (2008–2017)

		Year			Year		
		2008	2017		2008	2017	
		Males ASMR (95% CI)		APC (95% CI)	Females ASMR (95% CI)		APC (95% CI)
Diseases of the circulatory system	Urban	1597 (1473–1728)	2007 (1868–2153)	1% (−1.5–3.5)	1002 (911–1099)	1205 (1105–1311)	−0.3% (−3.9–3.4)
	Rural	1163 (1006–1337)	1477 (1299–1672)	3.7% (0.9–6.6)*	1117 (965–1287)	984 (842–1144)	−0.5% (− 3.9–3)
	Maritime	1828 (1377–2380)	1164 (810–1618)	−4.6% (− 8.8 – − 0.1)*	1474 (1037–2031)	796 (487–1230)	−8.9% (− 13.7 – − 3.7)*
Endocrine, nutritional and metabolic diseases	Urban	979 (882–1083)	950 (855–1053)	2% (− 3–7.2)	907 (820–1000)	979 (889–1076)	1.6% (− 3–6.4)
	Rural	445 (350–558)	617 (504–748)	5.4% (1.4–9.6)*	591 (481–717)	637 (523–768)	5.5% (0.2–11.1)*
	Maritime	731 (458–1107)	565 (329–905)	0.8% (− 6.7–8.8)	1155 (773–1659)	438 (219–784)	−4.1% (− 13–5.6)
Neoplasms	Urban	319 (265–381)	421 (358–491)	2.5% (0.3–4.8)*	372 (318–434)	548 (481–622)	3.6% (1.7–5.6)*
	Rural	125 (77–190)	285 (210–378)	11.7% (6–17.7)*	243 (175–329)	307 (230–401)	6.3% (− 0.1–13.1)
	Maritime	133 (36–340)	100 (21–291)	2.4% (− 6.5–12.3)	119 (25–349)	199 (65–465)	−4.2% (− 17–10.7)

APC: Annual Percentage Change; ASMR: Age-Standardised Mortality Rate; CI: Confidence Interval

*APC is statistically significant (*p* < 0.05)

### Focus group discussion results

Table 2 provides an overview of all themes identified during stage one of the analysis, by COM-B component. Sub-themes presented in **bold** text were those that generated most discussion, on which we carried out comparative analysis to identify gender differences and similarities. A narrative synthesis of findings is presented below, while Table 3 provides illustrative examples of quotes from the FGDs, by sub-theme.

**Table 2:** Themes emerging from the qualitative analysis of community focus group discussions, mapped to the COM-B model [38]. Themes generating most discussion are in bold text

CAPABILITY	OPPORTUNITY	MOTIVATION
***Physical***	***Social***	***Reflective***
Disability/ill-health impedes access to care for older adults	**Health worker attitudes and interactions with older adults**	Older adults’ not seeing the need for health care; acceptance of death
**Disability/ill-health restricts older adults’ ability to work and socialise**	Family/community attitudes and behaviour towards older relatives	**Family values and respect for older relatives directly influences quality of home-based care**
		Health worker altruism linked to proactive health care and social support
***Psychological***	***Physical***	***Automatic***
**Mental health affects well-being and care-seeking of older adults**	**Preference for community-based services that target the health care needs of older adults**	**Older adults’ fear of ageing, including loneliness, independence, financial and carer support**
Health worker knowledge and skills affects health care needs of older adults	**Accessible transport as a key to obtaining appropriate care**	**Dissatisfaction with the process of seeking care due to health worker attitudes and long facility wait times.**
**Family and carers’ knowledge/capacity to provide care for older adults**	**Poor access to essential medicines, mobility devices and health products.**	
**Older persons’ knowledge of available social and health services**	Health care facilities and services not responsive to the needs of older adults	
	Equipping carers to enhance healthy ageing	
	The link between an age-friendly environment and achieving good health in older age	
	**Impact of social welfare schemes on the quality of life of older adults and their families**	
	Policy environment lacks leadership and mechanisms to drive cross-sectoral coordination and implementation

**Table 3:** Illustrative quotes from community focus group discussions, by theme and gender

Comments by women	Comments by men
**CAPABILITY**	
**Knowledge and access to information**: *“Here, we just convey the message to one another. All of us receive social welfare, so if I hear something I just tell the other person. The departments don’t come here. Only if one of us visits their office and something new comes up then that person comes and tell us.”* **Disability**: “*My main problem is my physical health, especially my legs. … This makes it hard for me to go and receive my social welfare assistance – food voucher.”* *“For the older people that are sick, they can hardly work now and go to the farms. They are unable to gather food and earn money by selling crops.”* *“I had my leg amputated and I must use crutches and I paid $200 for the pair. Hospital never gave anything.”* **Family capacity:***“Taking care of older people is not an easy task. We need perseverance and a good heart to care for older people. There isn’t a lot of assistance from Government …”*	**Knowledge and access to information**: “*It is best the information is conveyed through the CHW.”* **Disability**: “*I used to go frequently to the farm, but I cannot do that now because I get short of breath whenever I do rigorous work. I thought it was asthma, but the doctor says my heart is weak.” “I must take a lot of medications and the side effects of the medications is a problem for me. It makes me drowsy and I get tired easily […] So, I cannot go out to visit friends.”* *“We have been working in the mines for a very long time, all of us here today. When you look at the two gentlemen here they are using canes due to wearing clogged shoes under the mines every day, every year. As for me my heart muscles are affected due to lifting heavy loads underground and it has affected me now on a long-term basis.”* **Mental health:** “*In good health means sleep well, eat sensibly, drink lots of water and have a lot of rest.”* *“It means eating good and nutritious food to stay healthy”* **Family capacity:***“I wish I was stronger so I can care for myself. I live with my son and his family. They look after me well but sometimes they cannot go for holidays because they worry about who will look after me.” “In families where their fathers are missing and [the fathers] are supposed to be earning for their families: these families usually have difficulties caring for their elderly relatives.”* *“Usually the women care for the elderly that are bedridden. They bathe and feed them. The women in the house do the most work.” “Okay, just imagine if in a family there is a young man in his twenties, and he is given the responsibility of looking after his elderly father, uncle or grandfather. This young man would not know how to responsibly care for him. He would need to cook and care for the elderly man and then go to his farm and try and earn a living off the farm. It is quite difficult.”*
**MOTIVATION**	
**Respect/ values:***“The issue now is that people are more focused on work and earning money. […] I feel that neglecting our cultural obligation to care for our elderly is becoming more apparent as the years go by.* **Fear of ageing:***“I see that if an older person doesn’t have money and did not have a good job when he [*sic*] was younger, their family will feel it’s a burden to look after them.” “For me I stay with my mother we sit down and share stories. Her only fear is being alone, now that she sees us around, she feels better.” “Yes, I worry about my health. And if I do get sick, who will look after me? I cannot always rely on my family. They also have their own lives to live.”* **Dissatisfaction with the process of seeking care:** “[It would be better] *if the elderly can avoid the queue altogether. It’s alright for those of us that are still fit, we can hold our stomachs and wait, but the elderly is not the same as us.”* *“They should attend to us first. We cannot wait for long. They treat us equally with other younger people that are not so sick. We are old, we cannot wait for long. When we come in and there are no chairs, none of the younger people stand so that we can sit and wait.”*	**Respect/ values:***“In this village, the elderly is respected. It is against our culture to have younger people mistreat the elderly. We try as much as possible to ensure that this occurs*.” **Fear of ageing:***“We sometimes worry that some of our children tend to neglect us due to our age.”* **Dissatisfaction with the process of seeking care:***“We aren’t as strong and fit as before to be able to stand for long hours and wait to consult a doctor”.* *.“So, we get tired and frustrated when we wait long hours and when we do finally get seen, our sugars are high and our pressure is high so the doctors then change our medication dosages and what not.”*
**OPPORTUNITY**	
**Family / Community attitudes:***“Before, families would collectively care for one another, but as people have moved to urban areas like Suva, they have tended to be less caring for everyone and become more individualistic.”* **Health worker attitudes:***I think they should learn to treat older patients more appropriately. The care they provide is good but it’s just the way they approach us. Maybe they are busy or tired.* **Transport: “***In this community, bus services are irregular and so we wait for a long time for a bus.” “Most times, the ambulance is also unavailable and when it does come, we must pay for it. There is a fee to use the ambulance.”* **Social welfare:***“When it started food voucher was $50 and till now it is still $50. There’s no change but things are getting expensive*.” *I rely on my social welfare money especially if I have to go for clinic, so if my clinic time does not coincide with the day I receive my social welfare money I end up not going to the hospital*.	**Family / Community attitudes: “***I don’t think we should see the care of our elderly as a burden. Many young people have begun to see it that way. With modern ideas we have started to lose focus on our culture and traditions of caring for the elderly.”* **Health worker attitudes:***“Some are good, and some are rude. I don’t want to sound like I’m being critical but there are some nurses that are very unfriendly and treat us harshly when we come to hospital.” “The older citizens are seen last, after normal rounds, [...] But we need rest.”* **Transport: “***Transportation is a problem and whenever the road is flooded it is very difficult for us to travel across.” “If you miss the 6:30 am bus, the next one is at 9:30 am and by the time you reach the hospital, your clinic time is over.”* **Access to medicines: “***Sometimes they just tell us to go the private pharmacy since they don’t have any of our medication at the hospital. We have to pay for our medications and not like before where we have access to free medication at the hospital pharmacy.” “We want quality medications that should be available all the time.”*

#### Capability

Knowledge of health and social support services was a key theme. Both men and women identified community health workers (CHWs) as their main source of information on health service availability. Women appeared better informed about health services availability and cited specific services and clinic times. Women said word of mouth (including from the village headman) was the most common means of receiving information on social welfare support; a number of women participants commented that neither government officials nor charitable organizations had visited their village. Men appeared to be better informed about government-run social welfare schemes, including  levels of financial support provided, and how to access them.

Men and women said that disability and ill-health, particularly lack of mobility, affected their autonomy, community and family engagement and ability to work. Women also mentioned the impact of disability on earnings, suggesting money was a front-of-mind concern, while men were more likely to comment on their physical capacity to perform work. Men were more likely to discuss the legacy of environmental and occupational health conditions on their health in older age.

Older men were more likely than older women to directly reference their mental or ‘psychological’ health, citing the importance of good diet, exercise and harmonious family relations to stay healthy. Women often expressed concern about the capacity of families to care for older people, the burden this placed on families and resulting family tensions. Women were more likely to recognize that caring for the elderly was hard work, likely a reflection that women typically perform the carers’ role.

#### Motivation

Both men and women spoke about the importance of values and respect for older persons and expressed a range of views on the extent to which these values were upheld. Women reflected on the care of an older person as a responsibility or a duty, while men – recalling their relationship as younger men with the previous generation – worried about the lack of respect they might receive from younger carers.

There was also sadness from both men and women that the older persons do not hold the same place in society as previously. Women feared ageing and were anxious about who would care for them as their health deteriorated, loneliness and financial insecurity. There was an awareness of these issues among women of all ages, with younger women recognizing that older women feared loneliness. Men were also concerned about ailing health and lack of family support but did not express concerns about loneliness or financial insecurity.

Men and women were consistent in expressing dissatisfaction with the process of accessing health care, with similar concerns raised in male, female and mixed focus group discussions. A key issue for both sexes was long wait times to access facility-based health care. Men also perceived that health staff were not sympathetic to their complaints and expressed lack of confidence in local (predominantly female) health workers. Women appeared more comfortable visiting community clinics. Men were more likely to go directly to hospital, despite their frustrations with long wait times.

#### Opportunity

All focus group discussions highlighted family and community attitudes towards older adults. The majority (men and women) commented that children and younger relatives were less willing and able to care for older persons, and women also noted families treated them differently as they aged, ‘especially when we don’t have husbands around.’ Changing social norms and loss of traditions were also mentioned, with some viewing their community as less cohesive and caring than in the past.

In relation to the physical environment, both men and women also raised concerns about access to medication – including lack of stock at public pharmacies and the need to purchase from private facilities. Lack of access to transport to visit clinics was also a concern: women were likely to mention the cost of transport, while both noted the challenge of travelling from remote areas to clinics. Women also noted crowded waiting rooms (nowhere to sit), and female carers of older persons commented on how the health system depended on their support. However, women expressed empathy with health staff, noting health centres were understaffed and under-resourced.

Finally, the low level of social welfare support and high costs of living and medication were prominent theme in the female focus groups. As with transport, women often cited the specific costs of items, again suggesting that making ends meet is a front of mind concern. While men did mention concerns over access to financial support and living costs, they did so much less frequently than women, and typically in more general terms.

### Policy analysis results

We identified 13 Fijian government policies, plans or strategies covering issues potentially relevant to the health and social care needs of older persons (see Table 4). Of these, three included objectives or commitments focussed on older women: National Policy on Ageing (NPoA, 2011–15); Fiji National Gender Policy (2014); and Ministry of Health Corporate Plan (2018–19). Table 5 contains *all* text from these documents on policy commitments or recommendations relevant to gender and ageing – in all cases this was very limited, with little detail on governance, financing, target setting or monitoring.

**Table 4:** List of Fijian Government Policies and Strategies relevant to the health care and social support needs of older persons

**Health & Disability.**	
Ministry of Health and Medical Services Strategic Plan 2016–20; Ministry of Health and Medical Services Annual Corporate Plan 2017–18; Ministry of Health and Medical Services Annual Corporate Plan 2018–19; Free Essential Medicines Program (2015). Fiji National Disability Policy (2008–2018); National Medicinal Products Policy (2013);	
**Support for older persons.**	
National Policy on Ageing (2011–2015); Decree establishing the National Council for Older Persons (2012);	
**Financial and social support.**	
Fiji National Financial Inclusion Strategy (2016–2020); Fiji National Provident Fund Decree No.52 (2011);	
**Gender.**	
Fiji National Gender Policy (2014);	
**National Development and Poverty Reduction.**	
Fiji Constitution (2013).	
Fiji National Development Plan (2017–2036).

**Table 5:** Policy measures targeting older women in relevant Fijian Government National Plans and Strategies

POLICY or STRATEGY	Gender focus
Fiji National Gender Policy 2014	*“Ensure development programs are inclusive of actions to assist elderly women, widows, and single mothers who are highly vulnerable to social economic pressures or disasters and who may have a high risk of poverty related diseases.”* (Section 5.12, paragraph 9, p.22) *“Recognise the special security requirements of women and girls including the young and the elderly. Ensure safe, equipped, confidential facilities for the provision of health care services which have adequate lighting, safe pathways, and protective infrastructure are available and maintained.”* (Section 5.12, paragraph 10, p.22)
Ministry of Health and Medical Services Annual Corporate Plan 2017–18;	This text is from Annex 7 of the Corporate Plan, which provides a matrix showing links between Corporate Plan targets and the Sustainable Development Goals. *“Provide quality preventive, curative and rehabilitative health services responding to the needs of the Fijian population including vulnerable groups such as children, adolescents, pregnant women, elderly, those with disabilities and the disadvantaged.”* (Targeted outcome, page 15) [Commitment repeated in Fiji’s Annual Operational Plan, 2018–19] *“Adopt and strengthen sound policies and enforceable legislation for the promotion of gender equality and the empowerment of all women and girls at all levels.”* (Targeted outcome performance measure, page 17)
National Policy on Ageing 2011–2015	*“Particular attention needs to be paid to older women because they make a up a larger proportion of the older population and are much more likely to be widowed, neglected, and poor.”* Policy summary, p. v *Develop awareness raising campaigns of the need to protect the rights of older persons, particularly the rights of older women and promote the use of key International Human Rights Conventions to which Fiji is a signatory, particularly the Universal Declaration of Human Rights and the Convention on the Elimination of All Forms of Discrimination Against Women.”* Goal 1, Objective 1, Strategy (i), p. vi *Support the care-giving role of older persons, particularly older women.* Goal 4, Objective 1, Strategy (iii), p. vii *“The Human Rights of older persons are respected and upheld, particularly of older women.”* Appendix 1: Implementation Matrix: Objective 1.2 p.2

When comparing these policy documents against the accepted pillars of policy development – such as problem identification, policy formulation implementation and evaluation [45] – further gaps emerge. Only the NPoA had a clear *problem analysis* in relation to gender and ageing. It recognized that women made up a larger proportion of the older population and that they were more likely than men to be disadvantaged, particularly in rural areas. It stated: “*Older women […] are likely to have lower education, less work experience, lower income and poorer access to assets than men, as well as diminished authority and autonomy within the family. Hence, women are more likely to be dependent, both upon the family and on public welfare programmes, especially at advanced ages and under conditions of illness and disability*” (p.6).

The NPoA’s *policy formulation* related in part to its own problem analysis, focussing on awareness raising of older women’s needs, and older women’s role as carers. However, it did not address the issues it identified of older women’s poverty and vulnerability, nor their higher burden of ill health and disability. Of four goals, 10 objectives and 21 strategies set out in the NPoA, only one goal and two strategies specifically mention older women. Further, there was no information on the process of *policy adoption* (how support for the policy will be secured) nor arrangements for implementation or monitoring.

The Fiji National Gender Policy did not articulate a process of problem analysis, policy development, or target setting in relation to older women’s needs. Rather, it mentioned older women in relation to a single issue: security. The Ministry of Health’s Corporate Plan encompassed older women in broad statements on gender equality and tailoring services to the needs of vulnerable groups but did not contain substantive information on how identified problems would be addressed.

Other relevant policies identified recognized either the needs of women, or the needs of older persons, but not *older women*. For example, the Fiji National Financial Inclusion Policy recognized that women were more likely than men to be excluded from financial services, such as not having a bank account, but the specific financial needs of older women were not mentioned. Similarly, the National Development Plan calls for improved data and evidence on the needs and poverty status of older persons, but not for disaggregated information on older women.

## Discussion

This study used three methods to investigate gender and ageing in Fiji: cause of death analysis; qualitative exploration of community attitudes to ageing; and, policy review. Our results show that applying a gender lens to the exploration of healthy ageing is feasible and insightful: we found that gender affects health outcomes among older persons, attitudes towards and experience of healthy ageing, and an older person’s access to and use of health services. We also found that Fiji’s policy response to health aspects of ageing has, to date, recognised the importance of gender, but taken limited action to address gender differences. Comparing information and data across the analytical streams, we identified four common themes or mutually reinforcing findings.

First, as elsewhere in the world, our findings suggest that gender has direct and indirect implications for the health of older Fijians. We found gender differences in mortality; expressions of mental health; and in knowledge, capacity and attitudes towards health care – which are in turn likely to impact health status. For example, we found that men have higher rates of cardiovascular disease (CVD) deaths while women have a high and growing death rates from cancer; that women were more likely than men to express feelings of fear, loneliness, anxiety; and that women have more knowledge of and confidence in primary care, which is likely to affect care seeking behaviour. Relevant policy frameworks in Fiji recognize that men and women have different health needs but do not consider in any detail how these affect health outcomes or care-seeking behaviour.

Second, gendered inequalities and patriarchal norms may underpin these differences. The gender differences in older people we identified reflect a cumulation of gender disparities across the lifespan, and in all aspects of Fijian society, not only provision of health care. UN studies suggest that elements of Fijian society are deeply patriarchal [26, 27] and a feminisation of poverty [46]. The high levels of concern we found among older women about their financial security is consistent with studies showing older women in Fiji are likely to be financially dependent on others [2]. Overall, Fijian women perform 52% of all work within the Fijian economy but receive just 26% of total income [47].

Our finding that men are more knowledgeable than women about how to access social support services confirms research showing Fijian women (of all ages) are less financially literate than men (16% less likely to have a bank account); older women are also less likely than older men to benefit from income support [26, 46]. The expectation that care of older persons be performed by female children or close relatives (older women themselves may also perform a caring role, when able) and older men’s anxiety about their declining capacity to perform physical work, also reflect gender norms that see women as responsible for ‘care’ and men for financial support.

Identified differences in mortality rates are consistent with gender-related lifestyle risks found elsewhere in the world (as opposed to sex-determined biological differences) which result in higher risk-taking behaviour [48] and higher CVD deaths among men [49].

Third, discriminatory gender norms may be influencing health and social care provision and policy. In health facilities, older men perceived that health staff were dismissive of their complaints and needs. Our policy analysis suggests that Fiji’s health and ageing policies largely fail to recognise the gender norms underpinning differences in health outcomes and found that the specific needs of older women are largely invisible in Fiji’s social care policies. This is not unusual in the Asia Pacific context, with UNFPA [50] noting: “*Despite discussion on the feminisation of later life in the Asia and Pacific region and calls for older women to be addressed specifically within national policy, very little gender specific policy was found […] in the countries reviewed. In most cases, one programme or one direct mention of older women in the area of social protection is the limit.”* That said, Fiji is one of only a few countries to include *any* mention of older women in national plans on ageing or related areas, suggesting it has a firm basis on which to build [50].

Fourth, gender inequality may be intersecting with age-discrimination, rural-urban inequities and intergenerational tensions. We found a disconnect between the traditional role of elders and their contemporary experience in Fiji, which may be particularly disempowering for men. As in many cultures, older persons expect to be treated with respect, and caring responsibilities are considered an inter-generational duty. However, attitudes are changing; we found men often voiced sadness about lack of respect from family members, while women were concerned about the impact on their financial security. In addition, men and women living in rural and maritime areas suffer the disadvantage of having poorer access to transport to health facilities.

Building on these four themes, we propose three broad recommendations to support the development of a stronger, gender-sensitive health system response to healthy ageing in Fiji:

1. **Develop a better understanding of the relationship between gender, (ill-)health and health service use** across all ages. This will help to identify the precursors and drivers of gender inequalities and ultimately improve quality of care for older persons. Early priorities for research based on our findings include identifying gender-related determinants of higher male CVD deaths: for example, masculinity norms that may drive risk-taking behaviour in men [48], leading to higher smoking rates [51]. Research is also needed to determine the causes of high cancer deaths among older women: do these relate to poor health literacy and/or to other barriers to cancer prevention and screening services, specific risk factors, or other reasons? A review of gender bias in service provision – to determine the level of priority afforded to conditions that predominantly affect women such as breast cancers – and in the attitudes of health staff may also be useful.
2. **Make health services accessible to and appropriate for older people of both genders,** for example by providing an age-friendly, safe, respectful and culturally-appropriate environment at health facilities, facilitating travel (or providing services close to home) and ensuring health workers have the appropriate attitudes and competencies [52]. Consultation with older persons and relevant stakeholders (such as the National Council on Ageing) should inform such changes.
3. **Ensure future revisions of key policies relevant to health and ageing set measurable targets** relevant to addressing gender inequities. This will help ensure that policy intent is translated to implementation.

### Strengths

Key strengths of our study were its comprehensive scope and mixed methods approach, drawing on qualitative and quantitative data, allowing us to illustrate the wide range of gender issues pertinent to healthy ageing in Fiji.

### Limitations

The key constraint was that our study was limited by the broader study on healthy ageing in Fiji, which for example determined the scope of the cause of death analysis and the focus of FGDs. The cause of death analysis was further limited by availability of data: Fiji uses ICD-10 coding and does not record any more specific information on underlying cause of death, so cause of death categories are relatively broad. The insights of health care workers, not reported in this study, would have further enriched our findings on older person’s use of health services. Future in-depth qualitative research ascertaining the views and priorities of health system planners and policy makers on health system responses to population ageing is warranted. Similarly, future research on the underlying reasons for the gender disparities is needed, and would support the targeting of policy, social and service interventions.

## Conclusion

This study demonstrates the feasibility and the importance of applying a gender lens to the study of healthy ageing. We found key gender disparities in health outcomes, the socio-economic and cultural aspects of ageing, and attitudes to health services, all of which have implications for policy and practice. Research on gender and ageing is scarce globally, but particularly limited in low- and middle-income countries, including in the Pacific. We anticipate our findings will be relevant beyond Fiji, to other Pacific island countries, and that the triangulation methodology may be an efficient and insightful way to examine gendered responses to healthy ageing.

## Supplementary Information

**Additional file 1: Supplementary File 1.** Age-Standardised mortality rates for Fijian men and women aged 55 and over, for top six causes of death.

## Data Availability

The data that support the findings of this study are available from the Ministry of Health and Medical Services (MHMS), Fiji but restrictions apply to the availability of these data, which were used under license for the current study, and so are not publicly available. Data are however available from the authors upon reasonable request and with permission of MHMS.

## Abbreviations

APC: Annual Percentage Change
ASMR: Age-standardised mortality rates
CHWs: Community Health Workers
CI: Confidence Interval
COM-B: Capability, Opportunity, Motivation, Behaviour
CRVS: Civil Registration and Vital Statistics
CVD: Cardiovascular Disease
FGDs: Focus Group Discussions
NPoA: National Policy on Ageing (Fiji)
UNFPA: UN Population Fund
WHO: World Health Organization
