# Supplementary material for: Gender, health and ageing in Fiji: a mixed methods analysis
Source: Int J Equity Health. 2021 Sep 14;20:205. doi: 10.1186/s12939-021-01529-9 (PMC8439064; doi:10.1186/s12939-021-01529-9)
Supplement: Supplementary file 1 — Additional file 1: Supplementary File 1. Age-Standardised mortality rates for Fijian men and women aged 55 and over, for top six causes of death. [file 12939_2021_1529_MOESM1_ESM.docx]

**Supplementary File 1: Age-Standardised mortality rates for Fijian men and women aged 55 and over, for top six causes of death**

|  | Age-standardised mortality rates (95% CI) | | | | | | | | | |
| --- | --- | --- | --- | --- | --- | --- | --- | --- | --- | --- |
|  | 2008 | 2009 | 2010 | 2011 | 2012 | 2013 | 2014 | 2015 | 2016 | 2017 |
| Diseases of the circulatory system | | | | | | | | | | |
| Male | 1483 (1386 – 1585) | 1793 (1686 – 1905) | 1934 (1823 – 2050) | 1622 (1520 – 1729) | 1637 (1535 – 1745) | 1809 (1701 – 1921) | 1814 (1706 – 1926) | 1720 (1615 – 1829) | 2023 (1909 – 2142) | 1814 (1706 – 1926) |
| Female | 1051 (973 – 1134) | 1303 (1216 – 1394) | 1485 (1392 – 1582) | 1173 (1091 – 1260) | 1030 (952 – 1111) | 1179 (1097 – 1266) | 1182 (1100 – 1270) | 1073 (995 – 1157) | 1307 (1220 – 1399) | 1129 (1049 – 1215) |
| Endocrine, nutritional and metabolic diseases | | | | | | | | | | |
| Male | 812 (740 – 888) | 721 (654 – 793) | 498 (443 – 559) | 978 (899 – 1062) | 993 (914 – 1078) | 966 (888 – 1049) | 886 (811 – 965) | 937 (860 – 1019) | 983 (904 – 1067) | 836 (763 – 913) |
| Female | 831 (762 – 905) | 721 (656 – 789) | 502 (449 – 560) | 1003 (927 – 1084) | 925 (852 – 1003) | 825 (756 – 899) | 962 (888 – 1042) | 891 (819 – 967) | 919 (846 – 996) | 866 (795 – 941) |
| Neoplasms | | | | | | | | | | |
| Male | 253 (214 – 298) | 283 (241 – 329) | 243 (205 – 287) | 329 (284 – 379) | 303 (260 – 351) | 327 (282 – 377) | 307 (263 – 355) | 324 (279 – 373) | 375 (327 – 428) | 367 (319 – 419) |
| Female | 328 (285 – 375) | 346 (302 – 395) | 323 (280 – 370) | 373 (327 – 423) | 393 (346 – 445) | 406 (358 – 458) | 393 (346 – 445) | 424 (375 – 478) | 441 (392 – 496) | 470 (418 – 526) |
| Symptoms, signs and abnormal clinical and laboratory findings, not elsewhere classified | | | | | | | | | | |
| Male | 281 (240 – 327) | 168 (136 – 205) | 192 (158 – 231) | 104 (80 – 134) | 183 (150 – 221) | 135 (107 – 169) | 154 (124 – 189) | 149 (119 – 184) | 170 (138 – 206) | 140 (112 – 174) |
| Female | 363 (318 – 413) | 206 (172 – 244) | 220 (185 – 259) | 147 (118 – 179) | 198 (165 – 236) | 153 (124 – 186) | 165 (135 – 200) | 151 (123 – 185) | 184 (152 – 220) | 164 (134 – 198) |
| Diseases of the respiratory system | | | | | | | | | | |
| Male | 183 (150 – 221) | 221 (184 – 263) | 197 (163 – 236) | 219 (183 – 261) | 218 (181 – 259) | 255 (216 – 300) | 298 (255 – 346) | 284 (243 – 331) | 257 (217 – 301) | 230 (192 – 272) |
| Female | 103 (80 – 131) | 112 (88 – 141) | 112 (88 – 141) | 101 (78 – 129) | 140 (113 – 173) | 106 (82 – 134) | 133 (106 – 164) | 114 (89 – 143) | 131 (105 – 162) | 120 (95 – 150) |
| Certain infectious and parasitic diseases | | | | | | | | | | |
| Male | 144 (115 – 178) | 185 (152 – 223) | 199 (164 – 238) | 168 (136 – 205) | 168 (136 – 205) | 166 (135 – 203) | 190 (156 – 229) | 176 (144 – 214) | 209 (174 – 249) | 176 (144 – 214) |
| Female | 145 (117 – 178) | 151 (123 – 185) | 168 (138 – 203) | 140 (113 – 173) | 144 (116 – 176) | 142 (114 – 174) | 198 (165 – 236) | 175 (144 – 210) | 164 (134 – 198) | 168 (138 – 203) |

CI: Confidence Interval
